# Spontaneous sparse learning for PCM-based memristor neural networks

**DOI:** 10.1038/s41467-020-20519-z

**Published:** 2021-01-12

**Authors:** Dong-Hyeok Lim, Shuang Wu, Rong Zhao, Jung-Hoon Lee, Hongsik Jeong, Luping Shi

**Affiliations:** 1 Department of Precision Instrument, Center for Brain Inspired Computing Research, Tsinghua University, 100084 Beijing, China; 2 Department of Electronic Engineering, Center for Brain Inspired Computing Research, Tsinghua University, 100084 Beijing, China; 3 Present Address: Department of Materials Science and Technology, Center for Future Semiconductor Technology, UNIST, 44919 Ulsan, South Korea

**Keywords:** Nanoscale devices, Electronics, photonics and device physics

## Abstract

Neural networks trained by backpropagation have achieved tremendous successes on numerous intelligent tasks. However, naïve gradient-based training and updating methods on memristors impede applications due to intrinsic material properties. Here, we built a 39 nm 1 Gb phase change memory (PCM) memristor array and quantified the unique resistance drift effect. On this basis, spontaneous sparse learning (SSL) scheme that leverages the resistance drift to improve PCM-based memristor network training is developed. During training, SSL regards the drift effect as spontaneous consistency-based distillation process that reinforces the array weights at the high-resistance state continuously unless the gradient-based method switches them to low resistance. Experiments show that the SSL not only helps the convergence of network with better performance and sparsity controllability without additional computation in handwritten digit classification. This work promotes the learning algorithms with the intrinsic properties of memristor devices, opening a new direction for development of neuromorphic computing chips.

## Abstract (web summary)

Designing energy efficient and scalable artificial networks for neuromorphic computing remains a challenge. Here, the authors present 1 Gb phase change memory memristor array with a spontaneous sparse learning scheme able to leverage the resistance drift issue improving the classification accuracy on MNIST handwritten digit dataset.

## Introduction

Recently, the field of artificial intelligence (AI) has witnessed tremendous advances in deep neural networks (DNNs)^1–7^. In DNNs, as a connectionist approach to AI, knowledge is represented by a hierarchically distributed pattern of activation nodes and a large number of weights storing the connection strength between the nodes^8–10^. Since weights are trainable depending on some relevant data without explicit rules, ‘learning’ can be defined naturally^11^. However, to gain powerful generalization capabilities, DNNs usually have massive tunable weights, thus the network training and inference are such data-intensive that the computation efficiency is usually limited by the memory access^12,13^. These networks are highly amenable to computation via large and dense matrix–vector multiplications that can be highly parallelized^14,15^. This has led to tremendous opportunities for hardware acceleration.

First, DNN accelerators and neuromorphic chips were designed with the application-specific integrated circuits to alleviate the memory access bottleneck to speed up the computation^16^. However, using this design approach, the synaptic weights are still stored in random access memories rather than directly encoded by the states of emerging analog devices and computed at the memory locations. Such analog devices assembled systems enable fundamental Non-von Neumann scheme and are believed to achieve significant speedup and power reduction for on-chip implementation of DNNs^17–21^. One of the most attractive analog devices is the two-terminal memristor, such as phase change memory (PCM) and resistive random access memory (RRAM), which offers the advantages of high density, fast operation speed, and low-power consumption^22,23^. The weight elements of DNNs can be represented by the conductance of memristors, which can be programmed by update pulses or read by low-amplitude reading pulses. Therefore, the weights in memristor networks could be accessed and tuned locally, making it extremely suitable for the DNN hardware representation^24^. When implemented in the crossbar structure, memristor networks are ideal substrates that directly perform multiply-accumulate operations (MACs) at the weight locations^25^. This parallel computing property speeds up the training and inference of DNNs with significant low-energy consumption, providing a promising computing paradigm for neuromorphic computing systems^26–28^.

However, there is still a huge discrepancy between the ideal unrestricted weight change in DNNs and the actual conductance change in memristors, resulting in the imprecise encoding of network weights and the corresponding computation. The conductance change of memristors is not only non-linear, asymmetric, and precision-limited, but also stochastic because of the device-to-device and pulse-to-pulse variations^29–31^. These properties generally lead to non-convergence when training real memristor neural networks. A promising solution to this issue is developing a co-optimization design that combines device properties (hardware insights) with DNN training techniques (software insights), such as quantization, pruning, and sparsification. Several approaches have been reported to restore the properties of the ideal weight, which ensures more stable performance but results in inefficiency and impracticality^17,32,33^. Our studies indicated that an adequate learning scheme able to exploit the stochasticity in PCM and incorporate brain-inspired approaches to enhance DNNs is essential to overcome this issue^34,35^. At present, the development of memristors is still insufficient to meet the ideal requirements for DNN training. Comparing the emerging memories for synapse device, PCM exhibits advantages on on/off ratio, endurance, and retention than RRAM, and on/off ratio than MRAM^36^. In addition, PCM is more mature technology as it has been manufacturing on a foundry basis. The physical mechanism of PCM is also well understood, which facilitates IC design of chip products. Thus, it is very practical to overcome the disadvantages of PCM and exploit the high reliability and the suitability for DNN applications. However, a critical issue for realization of PCM-based memristor neural network is that even if the weights represented by the resistance are precisely tuned to the ideal weights, its most cumbersome feature, the resistance drift issue, still remains, which has not been considered in previous studies^31,37^. Resistance can deviate continuously from the initial intended values because PCM undergoes a spontaneous resistance increase due to the structural relaxation after amorphization^38^. The resistance drift not only causes a major reliability issue of PCM, but also exacerbates the imprecise encoding of network weights in memristors. The conventional approach is to minimize the resistance drift by implementing error correction codes or adding specific periphery circuits, such as customized circuitry for rapid iterative write, modulation coding for drift-resilient, coding to new readout schemes that are drift resilient^39^. However, such approaches result in significant increase of complexity and power consumptions, and decrease of throughput.

Here we built a 39 nm 1 Gb PCM-based memristor neural network and quantified its non-ideal characteristics, especially the unique resistance drift effect. PCM weights play a unique role in in-memory computations (e.g., measuring time stayed in a weight state and modifying weight non-linearly according to the time) but the resistance drift effect is usually thought to be a problem for application. In this work, we make use of the unique resistance drift effect to develop a new learning scheme for PCM-based memristor neural network, and a multi-layer perceptron (MLP) with 784-256-10 structure and 203,264 weights was built. It has been reported that resistance drift increases the effect of image classification in SNNs, but it is explained as an effect of improving the weight representation and has not been linked to a new learning method^39^. In some neuromorphic computing systems, weights encoded by memristors are binary-valued (+1 or –1) for feasibility and thus weights stay in either +1 or –1 until an update process. To embody such abstraction in PCM-based memristor DNNs, we utilize the power of spontaneity of PCM memristor, that is, the electrical properties of the resistance drift that can be described by power law using a defect model^40–43^. In this setup, we fill the gap between actual behavior and unrealistic expectation in a PCM-based memristor DNN accelerator. At the same time, we incorporate sparsification based on weight consistency in a very natural way, i.e., discriminating a PCM weight staying consistently in high resistance with spontaneous resistance increase. During training, the negative weight (–1) corresponding to the crystalline state decreases the weighted sum (i.e., sum of multiplications between each input and weight) but the positive weight (+1 + *δ*, *δ* ≥ 0) corresponding to amorphous state increases the weighted sum for the non-negative inputs. Because *δ* is getting larger as long as a weight remains in the positive state, therefore, a prior knowledge that the consistency of positive weights increases the final output values potentially is incorporated in this PCM-based memristor neural network. Interestingly, the final output is selectively increased when the prediction is correct. That is, the consistency correlates with accuracy improvement. Weight consistency map reveals that the new learning scheme system prevents weights from being stuck in the one of weight states during the whole training, which controls the discrimination of the one weight state from the other weight state, i.e., the sparsity. It is noticeable that the weight sparsity is changed differently between input-side weights and output-side weights. The consistency boosts the tendency of sparsity enhancement by increasing the number of negative input-side weights but suppresses the increase of the number of negative output-side weights. Thus, SSL manifests the negative contribution of some input data to the accuracy by increasing the sparsity and utilize the minimize uncontrollable negative contribution to the output. A possible outline of PCM-based memristor neural network system, device implementation, operation schemes, and solution to resistance drift even after training is carefully addressed.

Our approach not only improves the classification accuracy of the PCM-based memristor neural network on MNIST handwritten digit dataset from 89.6% to 93.2%, but also controls the weight sparsity differently between layers (+1.4% for the input-side layer of about 200k weights while –4.5% for the output-side layer of about 2.5k weights) without any additional computation and operation. The demonstration based on the statistical parameters extracted from a 1 Gb PCM array also indicates higher feasibility. Unlike the passive approach of suppressing the drift effect, our SSL promotes the fusion of computer-based neural algorithms with the intrinsic properties of memristor devices^44–46^. To our best knowledge, this is the first learning method leveraging the PCM resistance drift to improve the memristor-based neural network performance. This opens up a new path for the development of PCM-based memristor neuromorphic accelerators.

## Results

### Description of the working system

**Fig. 1:**
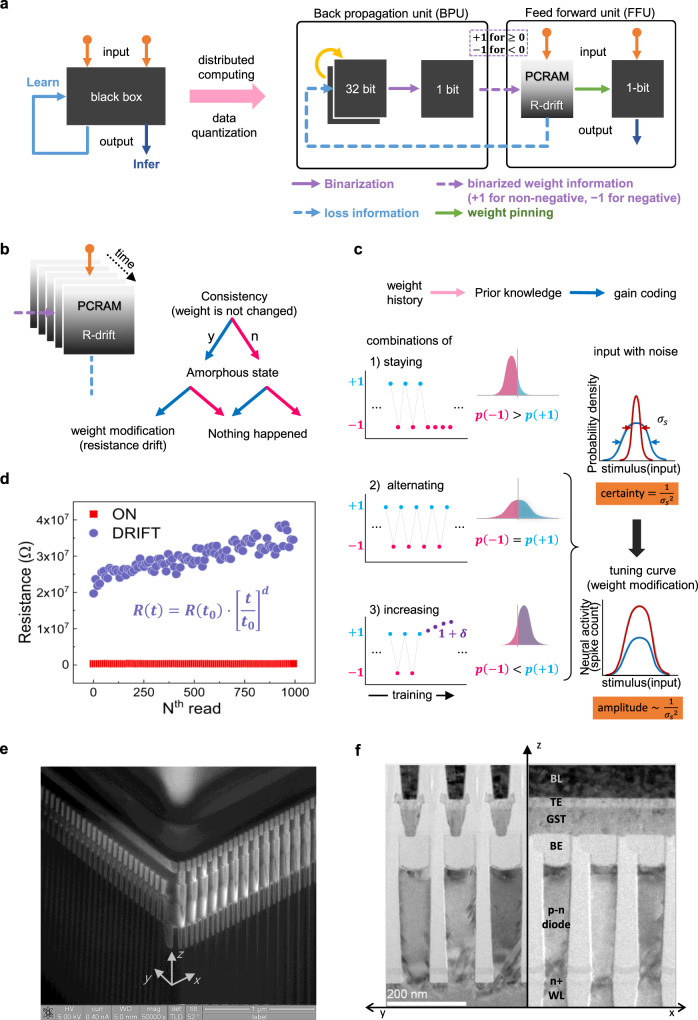
A PCM-based memristor neural network incorporating the resistance drift for the consistency-induced weight increase under distributed computing and weight precision reduction for new learning method. **a** The conventional neural network is depicted as a black box, which is divided into BPU (back propagation with 32-bit weight storage) and FFU (feed forward with R-drift incorporated PCM weight storage). The training process is completed with the PCM followed by the pinning operation to prevent unwanted R-drift for inference. **b** In conventional, weight change pattern does not affect itself directly. However, it can be possible through the resistance drift. For example, if a PCM remains in amorphous states consistently without switching, the weight is increased continuously according to the time. Then, weight change patterns can be reflected to weight value. **c** The high- and low-resistance are mapped to +1 and −1 weight state, respectively (see Fig. 2). The resistance can be in one of three states of ‘staying’ in crystalline state, ‘alternating’ between amorphous and crystalline, and ‘drifting’ in amorphous. Without priors about the weight, the weight will be +1 or −1 with 1/2 and 1/2 probability, respectively, in the conventional neural network. The probability that the final weight value is +1 is higher than 1/2 based on the likelihood in +1 state, like the Bayesian inference. The higher probability is encoded as greater weight value (1 + *δ*) like the gain coding in neuroscience, which indicates the increase of neural activity in amplitude as the input stimulus becomes certain. **d** Continuous resistance read shows different behaviors between amorphous (DRIFT) and crystalline states (ON). The resistance in amorphous is increasing gradually while the resistance in crystalline is almost constant. The resistance drift can be described by the power law. *R*(*t*_0_) is the first resistance measurement after the time *t*_0_. *R*(*t*) is resistance at time *t* (*t* > *t*_0_) and *d* is the drift coefficient which can be determined from a curve fitting. **e** SEM corner view of the PCM (39 nm, 1 Gbit) array used. **f** TEM images of some cells in the yz and xz planes reveal the damascene structure of GST layer.

Our approach is based on the distributed computing and the data quantization. A neural network is separated into several feedforward units (FFUs) and a central backpropagation unit (BPU) as shown in Fig. 1a^47^. BPU calculates weight modifications to update the old weights in high precision (e.g., float32) according to the feedforward output received from FFUs and supervised labels. Then the new weights are binarized in BPU and sent to FFUs. FFUs update their own PCM weight storage and send the loss for the next input to BPU again. In this configuration, the multiple FFUs share the computation workloads. Also, the data transfer from BPU to FFUs is reduced by the data quantization. After the training, the positive weights are pinned to a fixed value to be used for the inference without weight variation. BPU has its own full precision weight storage to be updated, like conventional neural network models for weight quantization. The weight is quantized and then stored in FFUs for the feedforward process. Although the storage is just replaced to PCM memristor in this neural network, the consistency is stored as additional resistance increase in PCM memristor automatically as shown in Fig. 1b.

A possible weight change in PCM memristor weight storage can be represented as a combination of three patterns; staying in –1, alternating between +1 and –1, increasing in +1 + *δ* (left column in Fig. 1c). The probability distributions of the corresponding full precision weights are simplified through the binarization (middle column). As it is binarized, it discards a lot of information about the full precision weight. However, Bayesian inference can be considered as a method to reconstruct information about the probability distribution of full precision weights, which predicts posterior probability based on prior probability and likelihood. Since it is not known how the full precision weight will be formed, +1, –1 can be thought of as having a prior probability of 50:50. This is a method of predicting the distribution of full precision weights based on likelihood where +1 and –1 appear when binarizing. We can infer that the distribution of full precision weights is shifted toward the positive side by reflecting the likelihood of +1. This inferred probability distribution is called the posterior probability distribution, and in the next inference, the posterior probability of the previous step becomes the prior probability, and then inference continues according to likelihood. There is no way to perfectly reflect this post-probability in a 1-bit memristor, but PCM can encode the increased probability with an increase in weight value using the drift effect. Then, according to the prior (i.e., the significance of positive weights), the posterior is determined by increasing the weights inspired from the gain coding (right column)^48,49^. Thus the weight increase based on the weight history can be rationalized by Bayesian inference and the gain coding. The resistance drift of a PCM memristor is compared after two different operations, crystallization, and amorphization. Continuous resistance read shows different behaviors between amorphous (DRIFT) and crystalline states (ON), as shown in Fig. 1d. The resistance of the amorphous state is increasing gradually according to the power law, while the resistance of the crystalline state is almost constant^41,42,50^. Thus, the prior can be incorporated into the weights naturally. Scanning electron microscopy (SEM) and tunneling electron microscopy (TEM) reveal the PCM array and the cell structure as shown in Fig. 1e, f (more details of PCM array and cell in “Methods” section)

### PCM memristor operation

**Fig. 2:**
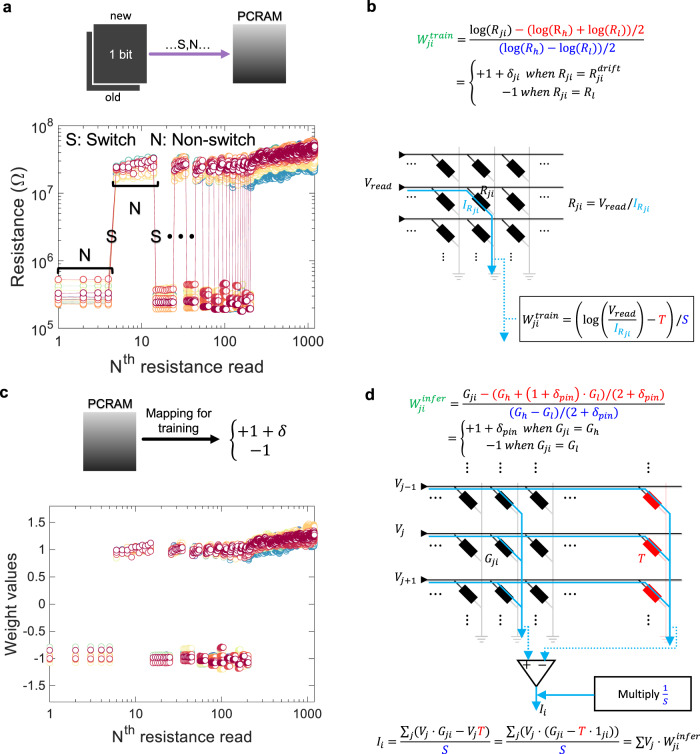
Mapping methods and corresponding circuits. **a** (Top) The schematic indicates the process of switching the PCM weight storage in FFUs according to the binarization of updated full-precision weight in BPU. (Bottom) A explanatory pattern of the resistance change is shown with corresponding sequence of S (Switch) and N (Non-switch). **b** (Top) The equation represents a mapping from resistance to weight. Instead of implementing MAC, resistance *R*_*ji*_ is read cell by cell in this mapping and the asymmetry in resistance variation (large variation in large resistance) is mitigated. (Bottom) The plot shows the results of the mapping from the resistance change in **a**. **c** (Top) The schematic indicates the mapping method defined in **b**. (Bottom) The resultant weight from the explanatory pattern of the resistance change in **a** through the mapping scheme in **b**. **d** (Top) The equation represents a mapping for the inference after the weight pinning process. (Bottom) A possible circuit to implement the feedforward acceleration (i.e., multiply-and-accumulate, MAC) is represented. The calculated results (*I*_*i*_) from the input (*V*_*j*_’s) in this circuit is identical to MAC process.

In our neural network, although the conventional weight storage is replaced with PCM memristor, the operation of the weight storage is the same as the conventional one. Some memristors are switched (S) and others are not (N). When a PCM memristor is not switched and remains in the amorphous state, the resistance is increased due to the spontaneous resistance drift. Figure 2a shows an example of how the resistance state of the PCM memristor changes as a sequence of specific S and N is delivered. The PCM memristor is switched occasionally in the middle of continual resistance read. The resistance read at the amorphous state shows a slight increase in resistance until it is switched back to the crystalline state. From 200 to 1200 resistance reads, the resistance drift continues without switching the amorphous state. In this way, the previously defined consistency in the pattern of weight evolutions is recorded as a resistance drift.

### Dual weight mapping strategy

To implement spontaneous weight modification from the resistance drift, we must consider how to map different physical quantities (e.g., high and low resistances) with positive values to typical weight values (e.g., +1 and –1, respectively). This can be solved by some practical mapping methods known as 2-memristor cell^31,45^ or dummy-cell methods^51,52^. Also, the mapping from conductance to weight values helps accelerate feedforward propagation, i.e., matrix–vector multiplication or MAC. However, training with resistance drift in conductance is not effective because some modifications are required to make the resistance drift effect equivalent in the conductance scheme. Therefore, we propose a resistance scheme for the training and conversion to conductance scheme for the inference.

For the training, the PCM memristor is written and read memristor cell-by-memristor cell, but is read as an analog memory in our practice. The computations of the feedforward propagation are shared with FFUs for several training data though MAC is not introduced. Thus, it is freer for processing the resistance values, e.g., taking the logarithmic value of the resistance to equalize the magnitude of the variations in high and low resistance (denoted by, *R*_h_ and *R*_l_, respectively). As shown in Fig. 2b, we obtained $W_{{ji}}^{\mathrm{{train}}}$ from the measurement of $I_{R_{ji}}$ and some arithmetic operations. The dummy-cell method was used to assign the weight increase property only to the positive weights. *T* denotes the translation (highlighted by red color) to make a pair of *R*_h_ and *R*_l_ centered at the zero point and *S* denotes the scaling (highlighted by blue color) to make the translated values +1 and –1, respectively. As a result, we can obtain the intended weight values as shown in Fig. 2c. Then, the weighted sum is calculated separately for the feedforward propagation for the training.

To accelerate the feedforward propagation during the inference after the training, it is required to map the weight values from the conductance. Because we propose the weight pinning, two distinguished static conductance states are required. Considering the reuse of the PCM memristor weight, all high resistance memristors (including resistance drift) are switched to low resistance (equivalently, high conductance denoted by *G*_h_) while all low resistance memristors are switched to high resistance (equivalently, low conductance denoted by *G*_l_). Then, *G*_h_ and *G*_l_ are mapped to +1 + *δ*_pin_ and –1, respectively. The mapping for the inference is similar to that for the training process except *T* and *S*. In this scheme, the resistance drift is negligible due to the reciprocal relation between the resistance and the conductance. To implement MAC, we just measure the total current *I*_*i*_ when adequate *V*_*j*_’s are supplied according to the input data. The measured current can be formulated from the circuit and the result is identical to the indented weighted sum as depicted in Fig. 2d.

### Classification results

**Fig. 3:**
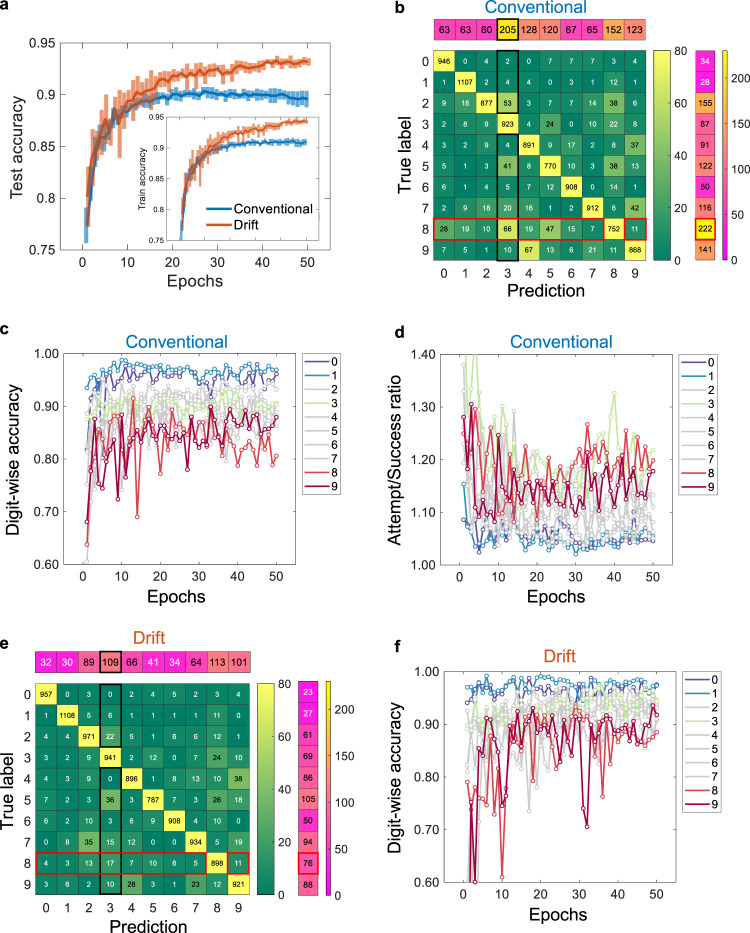
Classification accuracy. **a** The classification accuracy in the conventional and drift neural network is compared. The line is the mean value and the bar is standard deviation from 10 runs. Comparing with the conventional in both train and test accuracy, the drift exhibits smaller deviation in latter epochs while it does larger deviation in former epochs. The final test accuracies are 89.6% and 93.2% in conventional and drift, respectively. **b** Confusion matrix reveals the discrepancies between the true label and the prediction. The diagonal and off-diagonal components indicate the number of correct and incorrect predictions, respectively. The components in the additional row vector at the top and column vector at the right show the aggregated number of incorrect predictions along each column and true label along each row, respectively. The off-diagonal yellowish component indicates significant incorrect prediction in the conventional. The mostly mispredicted class is ‘8’ (red boxes) and the sum is 222. Also, the prediction as ‘3’ is mostly incorrect (black boxes) and the sum is 205. **c** Digit-wise accuracy reveals the difference of accuracy between digits. The digit ‘1’ seems to be mostly accurate while ‘8’ shows poor accuracy. **d** Digit ‘3’ and ‘8’ have top-2 attempt/success ratio of classification. However, the classification of ‘3’ is decent in the accuracy against the high attempt/success ratio while the classification of ‘8’ is not. **e** In the drift, the most misprediction of ‘8’ is mostly improved from 222 to 76. Also, the most misprediction as ‘3’ is mostly improved from 205 to 109, too. **f** Almost digits are improved in accuracy. Accuracy of digit ‘8’ and ‘9’ is greatly improved. Digit ‘0’ and ‘1’ still show high accuracy.

The most prominent result observed in this research is that the classification accuracy increased up to ~93.2% after the resistance drift effect was introduced. Figure 3a shows the training (inset) and testing accuracy (from 10 repeats) for the network with the conventional binary weight (denoted by ‘conventional’) and the network with the spontaneously increasing weight (denoted by ‘drift’). The remarkable fact is that the improvement in accuracy requires little additional computation cost and operation energy. Interestingly, drift demonstrated a larger deviation earlier, but a steady increase in accuracy with deviation reduced, unlike conventional (error bars) networks. This is because the consistency for the weight change could not be sufficiently assessed at the early stage of training and then the consistency is getting established and incorporated well.

To identify the improvement in accuracy, we examined the confusion matrix as shown in Fig. 3b and e. The confusion matrix indicated how many times the neural network classified each input digit image into each class. The diagonal number indicates correct classifications for each digit while the off-diagonal number means the total occurrence of the confusion (i.e., the discrepancy between the prediction and the truth). The numbers at the top and right (single row and column, respectively) are the sum of each row and column except diagonal value. For example, the highest value ‘205’ (sum of values surrounded by black lines in the confusion matrix) at the top means that the neural network mispredicted the input digit images as ‘3’ mostly (denoted by ‘misprediction as ‘3”), and the highest value ‘222’ (sum of values surrounded by red lines in confusion matrix) at the right means that the class ‘8’ is mostly mispredicted by the neural network (denoted by ‘misprediction of ‘8”). Because some specific digits like ‘3’ and ‘8’ showed distinctive results in confusion matrix, we compared a digit-wise accuracy during the training. The digit-wise accuracy is defined as the number of correct predictions divided by the number of actual images for each digit. Digit ‘0’ and ‘1’ outperform in accuracy while digit ‘8’ shows low accuracy overall in Fig. 3c. Although ‘3’ and ‘8’ show the top-2 highest attempt/success ratio (shown in Fig. 3d), the accuracy of ‘3’ is much higher than the accuracy of ‘8’. The improved result (denoted by ‘Drift’) shows that the number of misprediction as ‘3’ is reduced from 205 to 109 and the number of misprediction of ‘8’ is reduced from 222 to 76 significantly. The SSL shows accuracy improvement for most of the digits, especially better for ‘8’ as found in Fig. 3f.

### Drift effect during training

**Fig. 4:**
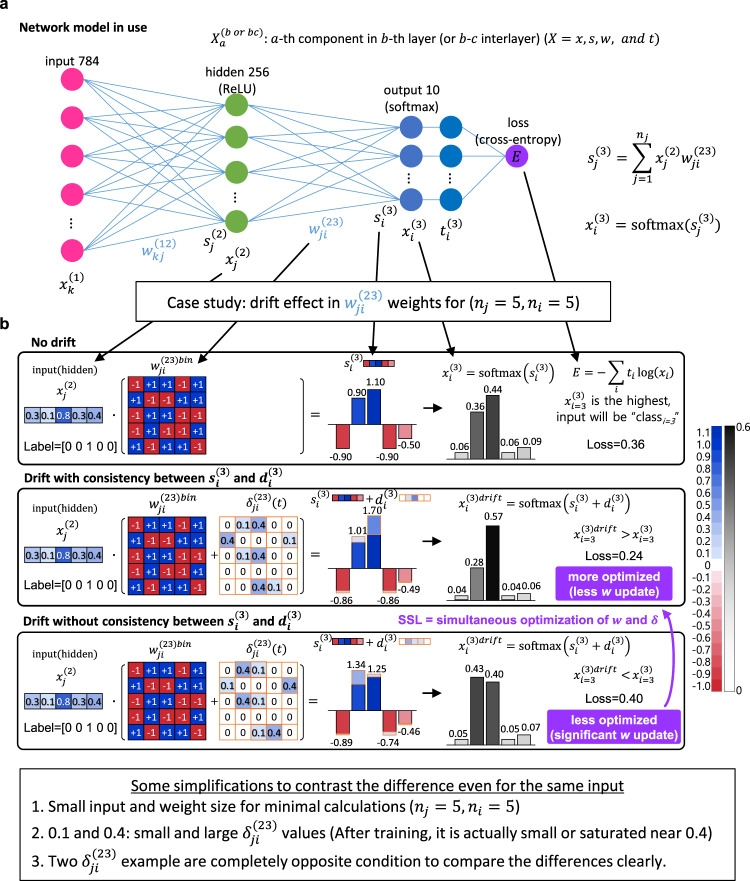
Feedforward explains the relation between the weight increase and the output values. **a** The network model in use is schematically depicted. The components *x, s, w*, and *t* denote for input, weighted sum, weight, and target, respectively. **b** Three different cases are compared to identify weight increase effects on the final output with some simplifications. All parameters are identical except the pattern of the weight increase term, $\delta _{ji}^{\left( {23} \right)}(t)$. The first panel explains the conventional feedforward process without the consistency-induced weight increase. The only weights between the hidden and output layers are considered. The input, weights, and the output are 1 × 5 row vector, 5 × 5 matrix, and 1 × 5 row vector, respectively in this schematic for convenience. The feedforward process can be calculated straightforwardly. For the input and weights, the final $x_{i = 3}^{\left( 3 \right)}$ has the greatest value and thus the neural network classify the input as ${\mathrm{{class}}}_{i = 3}$. In this case, the loss is 0.36 if the true label is [0 0 1 0 0]. In the middle panel comparing with the bottom panel, $\delta _{ji}^{\left( {23} \right)}(t)$ has the opposite pattern, i.e., the highest (0.4) and the lowest (0.1) values are swapped. According to the $\delta _{ji}^{\left( {23} \right)}(t)$, $d_i^{\left( 3 \right)}$ is added to the original $s_i^{\left( 3 \right)}$ in **a**, so some components are increased or decreased.

To reveal two contradicting results related to the output node ‘8’, that is, the weakest drift effect and the greatest improvement in accuracy, the impact of the drift effect on the final output in the feedforward process is analyzed in Fig. 4. Here, the actual 256 nodes in the hidden layer and 10 output nodes were simplified to five (*n*_*j*_ = 5, *n*_*i*_ = 5) for convenience as shown in Fig. 4a. Consequently, there are five classes (e.g., hand-written digits from 0, 1, 2, 3, and 4) to be distinguished and the weight has a 5 by 5 dimension. We introduced the drift effect as an additional term, $\delta _{ji}^{\left( {23} \right)}\left( t \right)$ to the weight connecting *i*th node in the second (hidden) layer and *j*th node in the third (output) layer. It should be noted that $\delta _{ji}^{\left( {23} \right)}$(*t*) is non-zero positive only for *j* and *i* satisfying $w_{ji}^{\left( {23} \right){\mathrm{{bin}}}}$ = +1. In this schematic, the input vector $x_j^{\left( 2 \right)}$ and weight matrix $w_{ji}^{\left( {23} \right){\mathrm{{bin}}}}$ are identical to those in other cases, respectively, but $\delta _{ji}^{\left( {23} \right)}$(*t*) is different for all the cases. We compared three different cases as shown in Fig. 4b. Among weights with a value of +1 in the weight matrix, the weight with a drift value of 0 indicates the PCM cell that has just switched to +1 state without time to drift. Weights with zero drift were arbitrarily selected. Those positive weights that are just switched will have various drift values. The top panel is a conventional drift-less feedforward process so the weighted sum and the softmax output are calculated as $s_i^{\left( 3 \right)} = \mathop {\sum}\nolimits_j {x_j^{\left( 2 \right)}} w_{ji}^{\left( {23} \right){\mathrm{{bin}}}}$ and $x_i^{\left( 3 \right)}$ = softmax($s_i^{\left( 3 \right)}$), respectively. Because $x_i^{\left( 3 \right)}$ has the highest value (0.44) at *i* = 3, the conventional neural network infers that the input is ${\mathrm{{class}}}_{i = 3}$. The middle and bottom panels indicate different effects of $\delta _{ji}^{\left( {23} \right)}$(*t*), and thus weights experience the drift differently. For instance, some elements of the weighted sum are increased or decreased according to which weights experience the drift effect greater or lesser. The effect of drift to be emphasized is that the change in each $s_i^{\left( 3 \right)}$ had a direct influence on every $x_i^{\left( 3 \right)}$ because of the mutually exclusive property of the softmax function. In the middle panel, large $d_{i = 3}^{\left( 3 \right)}$(=1.70−1.10 = 0.6) made $x_{i = 3}^{\left( 3 \right){\mathrm{{drift}}}}$(=softmax($s_{i = 3}^{\left( 3 \right)}$ + $d_{i = 3}^{\left( 3 \right)}$) = 0.57) larger than $x_{i = 2}^{\left( 3 \right)}$(=0.44) and $x_{i = 2}^{\left( 3 \right){\mathrm{{drift}}}}$ (=softmax($s_{i = 2}^{\left( 3 \right)}$ + $d_{i = 2}^{\left( 3 \right)}$)=0.28) less than $x_{i = 2}^{\left( 3 \right)}=0.36$ even for non-zero $d_{i = 2}^{\left( 3 \right)}$ (=1.01−0.90 = −0.11) simultaneously. Therefore, loss ($E = - \mathop {\sum}\nolimits_{i = 1}^{n_i} {t_i\log \left( {x_i} \right)}$) will be reduced from 0.36 to 0.24 in the middle panel because the index *i* of the largest $d_i^{\left( 3 \right)}$ is 3, which is identical to the index *i* of the true label of the input (on the other hand, $\delta _{ji}^{(23)}\left( t \right)$ has the opposite (0.4 and 0.1 were swapped from the middle panel) pattern in the bottom panel, so the prediction is incorrect (i.e., the indices are not identical each other) and the loss increases to 0.40.

Because $\delta _{ji}\left( t \right)$ is determined internally during the training process, we can only estimate it heuristically through the above two extreme cases. If the drift effect improves the prediction, the loss will be reduced and the weight update becomes smaller than the conventional case. On the contrary, if the drift effect makes the prediction worse, the loss will increase and the weight update becomes larger than the conventional case correspondingly. In this way, some weights that have a significant contribution to minimizing the loss function will most likely experience the drift effect.

### Weight consistency

**Fig. 5:**
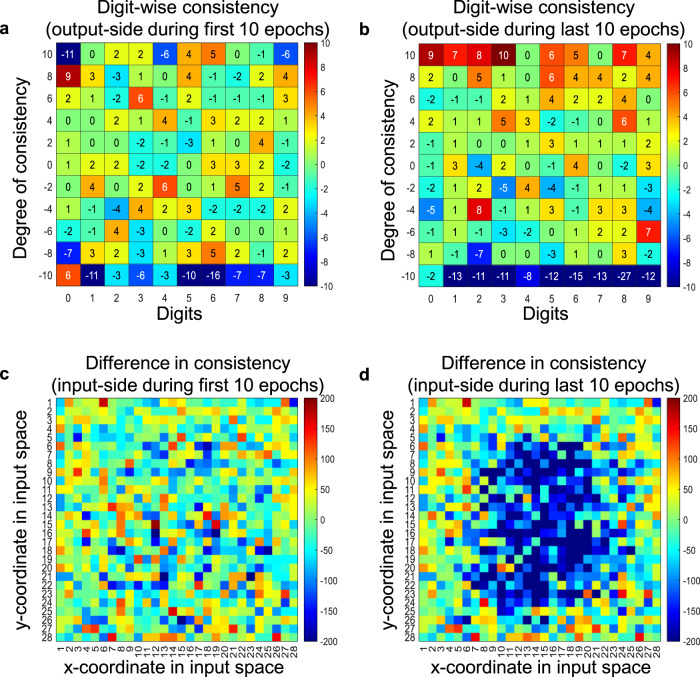
Weight consistency. **a** The graph explains how the drift effect affects the output-side weight consistency. The numbers in horizontal axis indicates the digit (0~9) to be classified. The numbers in vertical axis (−10 to +10) means how many times a weight stays in the same state for the first 10 epochs. Thus, all of 256 weights assigned to each digit fall into one of the values between −10 and +10 for both the conventional and drift networks. The values in the heatmap is the difference between the conventional and drift. The negative values at the bottom (deep blueish squares) indicates the slight reduction of the number of weights stuck in negative states. **b** During the last 10 epochs, the negative consistent weights is significantly reduced and some weights remain in the positive states long instead. **c** For the input-side weights, the consistency of 28 × 28 weights connected to each node in hidden layer of the neural network is accumulated. There is no noticeable pattern during the first 10 epochs. **d** During the last 10 epochs, some weights connected to the central region of the input images show stronger negative contribution to the accuracy.

Although it is hard to interpret the optimization of the drift effect during the training explicitly, the weight consistency reveals the pattern of weight changes. Figure 5a shows the results made from the subtraction of two heatmaps which implies the weight consistency in the conventional and drift, respectively. Thus, larger values indicate more significant drift effects on the weight consistency. The numbers in the horizontal axis indicates the digits (0–9) to be classified. The numbers in vertical axis (−10 to +10) denote how many times a weight stays in the same state for the first 10 epochs. The 256 weights can be considered to be associated with the output node ‘0’. The consistency of these weights can be reflected by adding each weight value once for each epoch. For example, if a weight has been in the −1 state for 10 epochs, the added weight value is −10. Thus, all of 256 weights assigned to each digit falls into one of the values between −10 and +10 for both the conventional and drift networks. The negative values at the bottom (deep bluish squares) indicates the slight reduction of the number of weights stuck in negative states. During the last 10 epochs, the negative consistent weights are significantly reduced, and some weights remain in the positive states long instead in Fig. 5b. For the input-side weights, the consistency of 28 × 28 weights connected to each node in hidden layer of the neural network is accumulated. There is no noticeable pattern during the first 10 epochs, but some weights connected to the central region of the input images show stronger negative contribution to the accuracy during the last 10 epochs as shown in Fig. 5c and d. At the early of training, there is no significant difference in weight consistency. As the training progresses, some weights stuck in either positive or negative states are remarkably reduced and some weights changing between two states are increased. Interestingly, the input-side and output-side weights show opposite patterns. Some weights stuck in the positive state are reduced for the input-side weights but some weights in the negative state are reduced for the output-side weights. Therefore, the accuracy-oriented consistency leads to the perturbation of some weights.

### Sparsity

**Fig. 6:**
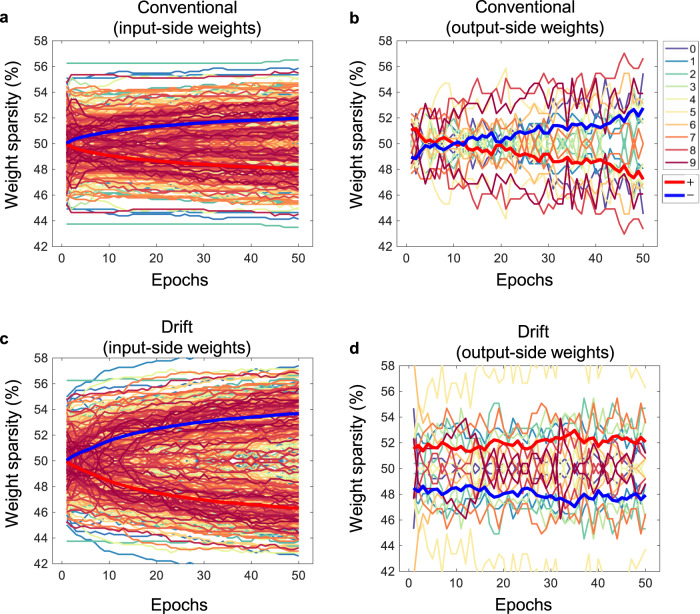
Weight sparsity. **a** For input-side weights, the number of negative weights is increased in the conventional. **b** For output-side weights, the sparsity shows the gradual increase of negative weights in the conventional. **c** The negative contribution is promoted in the drift. **d** The sparsity is fluctuated slightly and not changed significantly in the drift. Thus, weight sparsity exhibits different trends between output-side and input-side weights.

Because the perturbation of some weights is not reflected in the accuracy calculated at a certain time, the weight sparsity (i.e., the number of negative weights over the number of total weights) is examined. In the conventional, the number of negative weights is increased for both the input-side (Fig. 6a) and output-side (Fig. 6b) weights during the training. On the contrary, in the drift, the number of negative weights is further increased for the input-side weights (Fig. 6c) by about 1.4%, but the number of negative weights is decreased for the output-side weights (Fig. 6d) by about 4.5%. The blue and red lines indicate the proportion of the total negative and positive weights, respectively. The output-side weights are grouped by the output digits and the input-side weights are grouped by each node in hidden layers (i.e., 256 groups and 28 × 28 weights per group).

Considering the introduction of the accuracy-oriented consistency, the consistency of some weights compromising the accuracy will be excluded mostly. We can imagine an overlap between the consistency and accuracy. The weights can be subdivided by the position in the neural network (i.e., input-side or output-side), the consistency, and the sparsity. Therefore, the overlap portion (denoted by selectivity) determines the effectiveness of SSL scheme. Because the drift effect increases the output due to the non-negative contribution to the weighted sum basically, the similarity of the output vector to a one-hot vector indicates the effectiveness of SSL.

### Selectivity

**Fig. 7:**
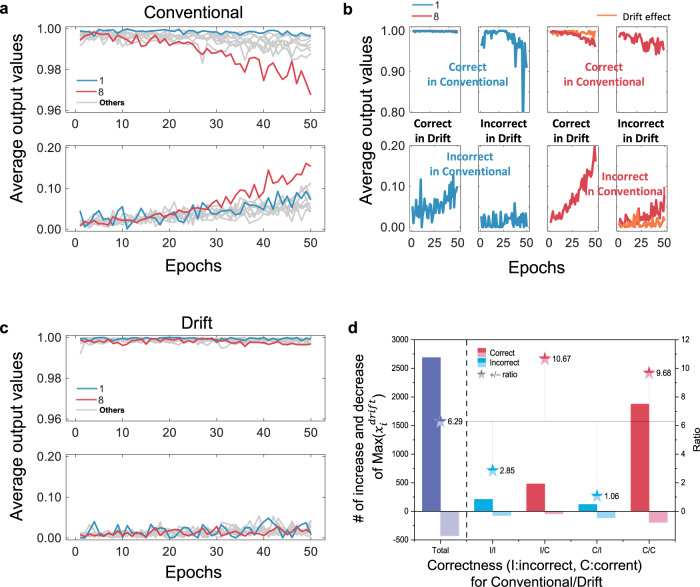
Selectivity. **a** Average output for the test image is compared among digits. The results show the greatest deviation from 1 and 0 for the digit ‘8’, which indicates ‘8’ is hard to train in conventional. Digit ‘1’ is the closest to 1. **b** Although the average output of ‘1’ is close to 1 in **a**, however, drift effect discriminates the ‘0’ input images two subgroups. The output of ‘1’ in the conventional is totally different according to whether the input image is classified correctly in the drift (two top-right bluish lines). That is, the output of ‘1’ in the conventional is extremely deviated from 1 for some images classified incorrectly in the drift. However, digit ‘8’ do not show the dependence on the correctness in the drift (two top-left reddish lines). The drift effect makes the output of ‘8’ close to an one-hot vector eventually (orange lines). **c** Average output is improved and then shows the promising output like a one-hot vector. **d** Number of increases and decreases of the maximum values of $x_i^{(3){\mathrm{{drift}}}}$ with respect to $x_i^{(3)}$ and their ratio demonstrates the selectivity of the output modification. The overall average is 6.29. When the drift neural network predicts correctly, the ratio is increased to 10.67 (for the test images predicted incorrectly in the conventional neural network) and 9.68 (for the test images predicted correctly in the conventional neural network). In the contrary, the ratio is far below the average when the drift neural network predicts incorrectly.

The maximum component in an output vector is <1 and the rests are larger than 0 as shown in Fig. 7a. We can define the digit-wise difficulty of classification based on the output vector, because the neural networks try to make the output like a one-hot vector as much as possible. The classification of a digit can be considered much difficult as more as the component of the digit in output vectors deviates from 1 or 0. In this perspective, the digit ‘8’ is much difficult to be distinguished from others than ‘1’ in the conventional. Surprisingly, the drift effect discriminates the digit ‘1’ output as two subgroups, though the average output of ‘1’ is close to 1 in Fig. 7a. In each group, the output of ‘1’ in the conventional is totally different according to whether a certain digit ‘1’ input image is classified correctly in the drift or not (two top-right bluish lines) as shown in Fig. 7b. That is, the output of ‘1’ in the conventional is extremely deviated from 1 for some images classified incorrectly in the drift. However, digit ‘8’ does not show such dependence on the correctness in the drift (two top-left reddish lines). The drift effect makes the output of ‘8’ close to a one-hot vector eventually (orange lines). In Fig. 7c, the average output is improved and then shows the promising output like a one-hot vector. The accuracy-oriented consistency subdivides both the digit ‘8’ and ‘0’ according to the difficulty. Then, SSL exploits the decision boundary of classification to maximize the accuracy.

The number of increases and decreases of Max($x_i^{{\mathrm{{drift}}}}$) compared with Max(*x*_*i*_) are plotted with respect to the correctness, as shown in Fig. 7d. The total number of increases or decreases can be divided into four cases, I/I, I/C, C/I, and C/C, where I and C denote ‘incorrect’ and ‘correct’ respectively, and the former and the latter refer to ‘conventional/drift’. The total number of increases is larger than that of decreases essentially and the ratio (i.e., the number of increases over the number of decreases) is 6.29. It is noticeable that the ratio is remarkably higher when the classification of the drift case is correct. Simultaneously, the ratio is reduced considerably when the result is incorrect. The ratios are 10.7 and 9.68 for I/C and C/C (above the overall ratio), whereas they are 1.06 and 2.85 for C/I and I/I (below the overall ratio), respectively. The results indicate that the selectivity of the output increase is originated from the consistency and can be supported analytically by tracing the backpropagation process.

### Weight pinning

**Fig. 8:**
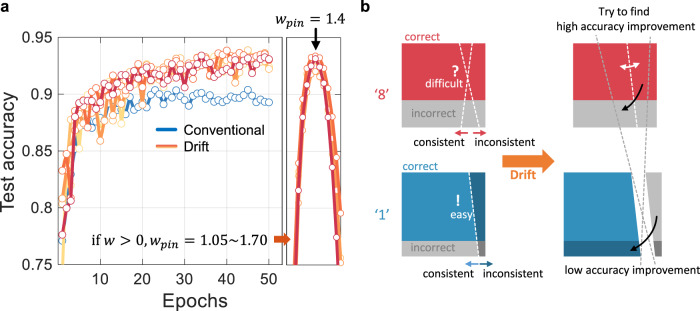
Weight pinning and summary. **a** Due to the spontaneity of the resistance drift, the weight should be pinned not to lose the learned from the consistency. After the training, we can find a suitable weight value *w*_pin_ (e.g., by scanning from 1.05 to 1.70) to maximize the accuracy near the best obtained for the training. *w*_pin_ is 1.4 for the neural network and corresponding parameters used in this work. **b** The effect of the consistency-induced weight increase can be summarized as a weight sparsification based on the input-wise classification difficulty.

One important issue of the spontaneous weight modification utilizing resistance drift is that the optimized weight will undergo inevitable further change after training. However, a suitable constant weight value can be found to represent all individually changed weights due to the drift without degrading performance in the inference process. After training, all positive weights can be regarded as a fixed positive constant like a conventional digital memory device. Then we can find the constant that maximizes the accuracy of the test images. When the fixed positive constant is changed from 1.05 to 1.70 by 0.05 step, we compared the accuracy and then found that it is compatible to the accuracy just after the training when *w*_pin_ = 1.4 (name after weight pinning) as shown in Fig. 8a.

## Discussion

From the digit-wise analysis, we found some results. (1) Digit ‘8’ classification is mostly inaccurate. (2) The accuracy-oriented consistency (i.e., drift effect) is introduced. (3) The consistency controls the weight sparsity, so the weights are regrouped based on the digit-wise (via output-side weights) and image-wise (via input-side weights) manners. (4) By drift effect, digit ‘1’ input images are easily subdivided into two parts while digit ‘8’ input images are not. (5) Then, the network increases the classification accuracy of ‘8’ mostly by exploiting the room for weight modification without degrading the classification accuracy of ‘1’. The accuracy improvement due to consistency-induced weight increase is summarized as shown in Fig. 8b.

Our approach is rationalized in that the difficulty of classification is related to the difficulty of weight determination (i.e., consistency)^53–55^. The difficulty-based accuracy improvement is similar to the experimental results demonstrating the decision confidence in the brain^56–59^. In animal experiments, different neural activity according to the difficulty of the decision is the most important feature of the confidence^49,60^. For example, for a mixture of two stimuli (e.g., scent A and scent B) with a different ratio, neural activity is most active for incorrect decision in easy decision tasks (A: 100% +B: 0% or A: 0% +B: 100%) and least active for correct decision in easy decision tasks. In the contrary, for the decision in difficult decision tasks (A: 51% +B: 49% or A: 49% +B: 51%), neural activity is moderately active regardless of the correctness. For this reason, the process of confidence formation is to classify easy tasks definitely, which is similar to the classification of ‘1’ under the drift effect.

It is generally known that the reset current decreases as the cell size decreases^61,62^. In addition, a lower reset current is associated with a decrease in amorphous volume and consequent higher amorphous resistance^63^. Interestingly, the resistance in the amorphous state correlates with the drift coefficient^64^. The higher the amorphous resistance, the greater the drift coefficient. To our best knowledge, there is no publication on the impact of technology scaling on the resistance drift yet.

Various experimental results have explained resistance drift in different ways and share a commonality that it is related to structural relaxation and stress release. Therefore, the electronic state of the material has a direct effect on the resistance drift rather than the technology node scaling or device structure. When PCM cell is not fully reset, the drift coefficient as well as resistance becomes smaller. The drift coefficient of the fully reset GST is ~0.09–0.11^39^. In our case, PCM is almost fully reset (~0.10) and thus the impact due to technology node reduction is negligible. Even if the drift coefficient becomes greater with the size scaling or structure changes, we can slightly increase the reset current to decrease the reset resistance, thus maintaining the drift coefficient to a similar level.

We tested the classification accuracy over a wide range of drift coefficient means and deviations as shown in Supplementary Fig. 7. The consistently improved accuracy indicates that our learning scheme is very robust to the changes in drift effect caused by technology scaling or environmental temperatures. Interestingly, it is known that the drift coefficient does not depend on the constant temperature^42,65^. If the temperature rises suddenly, the drift coefficient temporarily increases. As shown in Supplementary Fig. 7c, the accuracy remains increasingly improved until the drift coefficient increases to ~1. Since the drift factor in the fully reset state is bounded, it does not have a value much higher than 0.12 for GST-based PCM^43,66^. Therefore, the accuracy of proposed scheme will not deteriorate at a high temperature as long as it is below the glass transition temperature of phase change materials.

In this work, we built an ensemble of a mature 1 Gb PCM array with 39 nm technology, and by leveraging the statistical parameters obtained from the measurement of resistance drift, demonstrated a spontaneous sparse learning scheme in a PCM-based memristor neural network. By encoding the consistency of weight changes into the weight increases spontaneously, the neural network demonstrates the discriminated weight modification depending on the classification difficulty. Without any additional computation and operation costs, this new training scheme with sparsity controlled (+1.4% for output-side weights and −4.5% for input-side weights) improves the accuracy (93.2%) of a handwritten digit classification application (MNIST dataset) compared with the baseline neural network (89.6%).

Additionally, due to the compatibility of the consistency-induced weight increase with any PCM-based memristor neural network, our results have potential to be related to evolutionary neural networks^4,5^ inspired by the spontaneous behavior and modular neural networks^6,7,67^. Our work provides a potential prototype neural network that implements a metacognitive function, such as confidence, with low computation workloads for lightweight devices. Furthermore, our approach can be applied to RRAM as well as PCM, and improved learning effects are expected in more complex networks using multi-level cells. Although the resistance drift is weak in RRAM, the resistance drift appears distinctly in the conductive-bridge RAM, one of RRAM branches, due to the lateral diffusion of the metal constituting the bridge^68^. Since the drift coefficient is roughly observed within the range of 0.001–0.1 similar to PCM depending on the resistance of the initial resistance, the method presented in this study is expected to be applicable to those RRAM devices^69^. Our work may provide inspirations to RRAM research, i.e., leveraging the unique material characteristics (e.g., conductive-bridge modulation) to improve the performance/efficiency of memristor networks or to incorporate another learning scheme into memristor networks. The basic operation of our PCM array is well evaluated up to the 2-bit 4 level. The proposed SSL algorithm is expected to further improve the performance of network in the case of multilevel operation.

Our proposed learning scheme utilizes the intrinsic resistance drift to improve the learning accuracy without additional cost, which opens up a new path to develop learning schemes using the materials’ properties.

## Methods

### Device fabrication

Cross-point PCM arrays of cells with *n*+-Si/n-Si/*p*-Si/TiN/Ge_2_Sb_2_Te_5_ (GST)/TiW/W structures were fabricated on a 300-mm Si wafer. A highly doped *n*+-Si layer was prepared with B at a doping concentration exceeding 10^22^ atoms/cm^2^. The *n*-Si/*p*-Si diode was epitaxially grown on the wafer by chemical vapor deposition (CVD) with B at a doping concentration of 10^21^ atoms/cm^2^ for *n*-type and P at a doping concentration of 10^20^ atoms/cm^2^ for *p*-type. TiN films were deposited on the diodes by physical vapor deposition (PVD) as the bottom electrode (BE). The stack was dry-etched by etching along the *y*-direction and then along the *x*-direction to form pillar structures. The stack of n-Si/p-Si/TiN was etched into *y*-axis-oriented parallel lines using a hard mask of SiO_2_. The gaps between the etched lines were filled with SiO_2_. The gap-filled structure was flattened by chemical–mechanical polishing (CMP) and etched again with *x*-axis-oriented parallel lines. This formed word lines (WL) of n+-Si at the bottom of the diode. The gaps between diodes were then filled with SiO_2_ and flattened by CMP. Then, interconnects and trenches were formed in the SiO_2_ with lithography along the *y*-direction to build GST and a top electrode (TE) on the TiN through the dual damascene process. The interconnects and trenches were filled with GST and then TiW was deposited as the TE. After CMP, a line of W metal was formed using lithography on the TiW as the bit line. Finally, the cross-point PCM array was fabricated. The contact area between the GST and bottom electrode was ~40 nm × 80 nm.

### PCM Array information

The PCM Array used in the research is an array with 39 nm technology in the process of developing PCM arrays with 90, 50, 45, 39 and 20 nm technologies^70–72^ (officially reported for some technology only). Various operations and evaluation data including temperature and resistance drift for each technology were partially disclosed^73,74^. For example, resistance distributions for four resistance levels of (00), (01), (10), and (11) after an elapse of 400 h (1.4 × 10^6^ s) and an additional thermal annealing at 130 °C for 12 h bake were provided for 90 nm PCM array. Also, for 45 nm PCM array, analysis of the drift effect was presented in depth to improve multi-level operational characteristics and verify practicality. The total array sizes fabricated by 90 and 45 nm technology are 512 Mb (16 banks of 32 Mb, 8 blocks/bank) and 1 Gbit (16 banks of 64 Mb, 8 blocks), respectively. For the 39 nm PCM Array, the temperature and drift characteristics were not officially disclosed at the wafer-level, but they are similar to those of the 45 nm PCM array. For the 39 nm PCM array, 2-PCM synapse behavior was evaluated^75^. Although the operation circuit was slightly deformed according to the characteristics of each technology to implement ancillary functions for performance improvement, such as high read or write throughput, the characteristics of each PCM array were well evaluated as can be seen in the literature provided.

### Measurement

Each PCM cell is in the low resistance state at about 10^4^ Ω which was measured by using 1 V pulse with 5 ns rising time, 60 ns duration, and 5 ns falling time. The cell resistance is increased to about 10^7^ Ω using a pulse of 5 V height with 5 ns rising time, 60 ns duration, and 5 ns falling time. We then make the cell have a few 10^4^ Ω by applying a voltage of 3 V with 5 ns rising time, 400 ns duration, and 300 ns falling time. The PCM cell is switched 20 times between set and reset states with 10 resistance read after each switching. Then the resistance is measured 1000 times just after the reset switching to measure the resistance drift as depicted in Supplementary Fig. 1a. A pulse generator (Keysight 33600A) was used to provide electrical pulse to the cell. A source meter (Keithley 2600) was used to measure the cell resistance. Pulse is applied to the TE while bottom electrode is ground through the contact pads. Supplementary Fig. 1b shows the wafer being measured.

### Parameter extraction

We extracted resistance distribution and deviation from over 100 cells. For each cell, the set and the reset resistance were measured over 100 data points and then the resistance drift was measured for 1000 data point with 1 read-operation per 1 s. Then, the sample mean and the sample standard deviation of the set resistance, the reset resistance, and the drift parameter (*d* in Eq. (1)) were extracted and denoted by $\mu _{{\mathrm{{SET}}}_i}^{\mathrm{{s}}}$, $\sigma _{{\mathrm{{SET}}}_i}^{\mathrm{{s}}}$, $\mu _{{\mathrm{{RESET}}}_i}^{\mathrm{{s}}}$, $\sigma _{{\mathrm{{RESET}}}_i}^{\mathrm{{s}}}$, $\mu _{{\mathrm{{DRIFT}}}_i}^{\mathrm{{s}}}$, and $\sigma _{{\mathrm{{DRIFT}}}_i}^{\mathrm{{s}}}$, respectively, for the $i{{\mathrm{th}}}$ cell. Then, the statistical parameter for the resistance distribution, i.e., population mean ($\mu _{{\mathrm{{SET}}}_i}^{\mathrm{{p}}}$, $\mu _{{\mathrm{{RESET}}}_i}^{\mathrm{{p}}}$, and $\mu _{{\mathrm{{DRIFT}}}_i}^{\mathrm{{p}}}$) and population standard deviation ($\sigma _{{\mathrm{{SET}}}_i}^{\mathrm{{p}}}$, $\sigma _{{\mathrm{{RESET}}}_i}^{\mathrm{{p}}}$, and $\sigma _{{\mathrm{{DRIFT}}}_i}^{\mathrm{{p}}}$) with an error term depending on the number of sampling data and the distribution model were calculated for the *i*th cell. Supposed that the resistance distribution is a normal distribution, $\mu _i^{\mathrm{{p}}}$ can be estimated as $\mu _i^{\mathrm{{s}}}$ with standard error of $\frac{{\mu _i^{\mathrm{{s}}}}}{{\sqrt n }}$ while $\sigma _i^{\mathrm{{p}}}$ can be estimated as $\sigma _i^{\mathrm{{s}}}$ with standard error of $\sqrt {\frac{2}{{n - 1}}} \left( {\sigma _i^{\mathrm{{s}}}} \right)^2$. This process is depicted in Supplementary Fig. 2 (i and ii) for *R*_*ij*_, *j*th measurement of resistance of *i*th cell. In addition, the cell-to-cell population mean (*M*^p^) and population standard deviation (*Σ*^p^) were determined for both distributions of $\mu _i^{\mathrm{{p}}}$’s and $\sigma _i^{\mathrm{{p}}}$’s as shown in Supplementary Fig. 3 (iii and iv), which are denoted by $M_\mu ^{\mathrm{{p}}}$, $\Sigma _\mu ^{\mathrm{{p}}}$ and $M_\sigma ^{\mathrm{{p}}}$, $\Sigma _\sigma ^{\mathrm{{p}}}$. By sampling a value from the normal distribution defined by $M_\mu ^{\mathrm{{p}}}$ and $\Sigma _\mu ^{\mathrm{{p}}}$, we could determine a mean resistance (μ^*^) of a possible PCM cell. Then, the resistance deviation (α^*^) that corresponds with μ^*^ is determined as a value that has the same cumulated probability in the normal distribution defined by $M_\sigma ^{\mathrm{{p}}}$, $\Sigma _\sigma ^{\mathrm{{p}}}$. Thus, six unique parameters ($\mu _{{\mathrm{{set}}}_i}^ \ast$, $\sigma _{{\mathrm{{set}}}_i}^ \ast$, $\mu _{{\mathrm{{reset}}}_i}^ \ast$, $\sigma _{{\mathrm{{reset}}}_i}^ \ast$, $\mu _{{\mathrm{{drift}}}_i}^ \ast$, and $\sigma _{{\mathrm{{drift}}}_i}^ \ast$) were assigned to each PCM cell for set, reset, and drift independently and randomly. Finally, $R_{{\mathrm{{set}}}}$, $R_{{\mathrm{{reset}}}}$, and *d*_drift_ were generated and renewed every switching based on the uniquely assigned parameters to PCM cells. This process is depicted in Supplementary Fig. 4 (v, vi, and vii) and key statistical data that is determined from measurement are represented in bottom of the figure.

### Neural network

We used a neural network with 784 nodes in input layer (denoted by $x_k^{\left( 1 \right)}$), 256 nodes in one hidden layer (denoted by $x_j^{\left( 2 \right)}$), and 10 nodes in output layer (denoted by $x_i^{\left( 3 \right)}$), for the MNIST hand-written digit classification, as shown in Supplementary Fig. 5. The layers and the weights between the layers are distinguished by the superscript. All nodes are fully connected and any regularization process such as batch normalization and dropout are excluded. Each 28 × 28 greyscale input image is normalized to [0, 1] and denoted by $x_k^{\left( 1 \right)}$. The weights are initialized by the variance scaling initialization. We applied ReLU activation in the hidden layer and softmax in the output layer, and then cross-entropy function was used as a loss function. To exclude any adaptive factor, we used stochastic gradient descent optimizer, constant learning rate of 0.001, and mini-batch size of 100. Because we propose PCM as a weight device, the full precision weights are binarized and then the results are written in PCM cells. Some PCM cells which are in amorphous state and not updated experiences the drift effect. The modified weights increase the weighting of some inputs and affects the whole training process. The algorithm is described in Supplementary Fig. 6.

## Supplementary information

Supplementary Information

## Data Availability

All the related raw data, trained parameters, and codes are available from the authors upon reasonable requests.
