# Supplementary material for: Spontaneous sparse learning for PCM-based memristor neural networks
Source: Nat Commun. 2021 Jan 12;12:319. doi: 10.1038/s41467-020-20519-z (PMC7803975; doi:10.1038/s41467-020-20519-z)
Supplement: Supplementary file 1 — Supplementary Information [file 41467_2020_20519_MOESM1_ESM.pdf]

Spontaneous Sparse Learning for PCM-based Memristor Neural Networks

Dong-Hyeok Lim[1,3], Shuang Wu [1], Rong Zhao[1], Jung-Hoon Lee[2], Hongsik Jeong[2,3,*], Luping Shi[1,*]

[1]Center for Brain Inspired Computing Research, Department of Precision Instrument, Tsinghua University, Beijing, China, 100084

[2]Center for Brain Inspired Computing Research, Department of Electronic Engineering, Tsinghua University, Beijing, China, 100084

[3]School of Materials Science and Technology, UNIST, Ulsan, South Korea, 44919 (current affiliation)

*corresponding authors: Hongsik Jeong (hsjeong1@unist.ac.kr) and Luping Shi (lpshi@tsinghua.edu.cn)

Supporting information

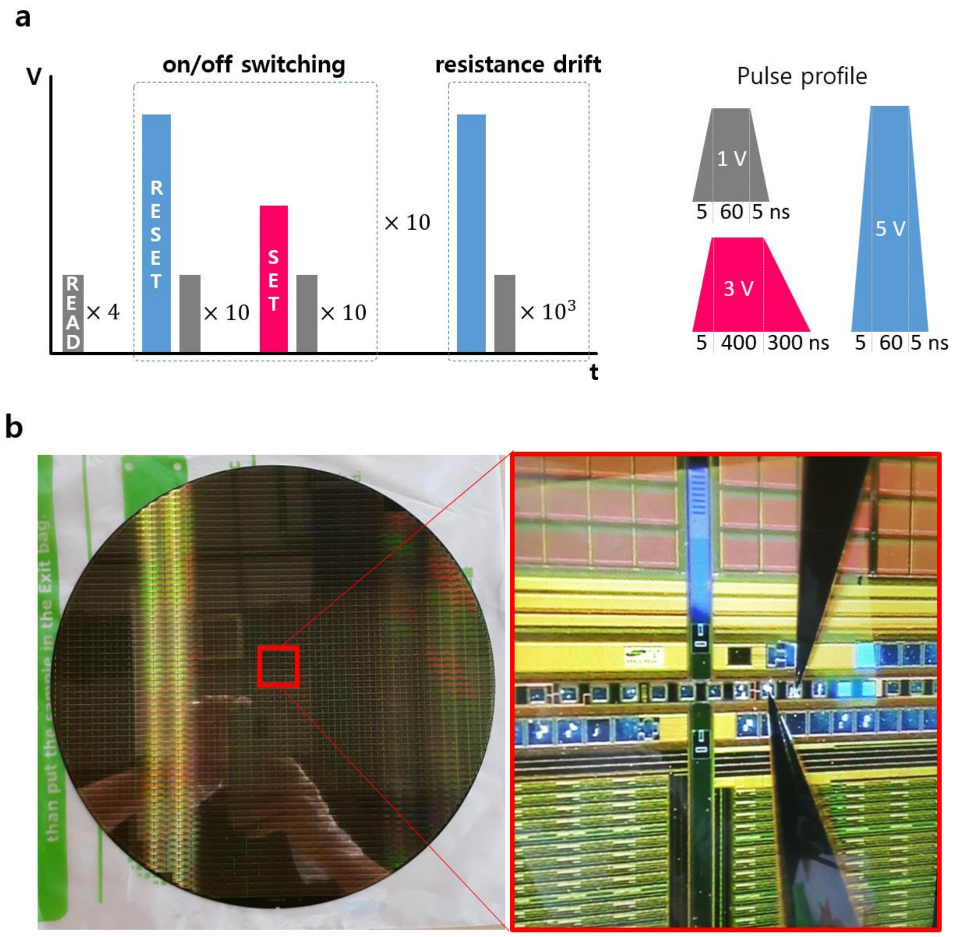

Supplementary Figure 1. a) The measurent conditions are depicted. b) The wafer used in this work and its magnified image are represented.

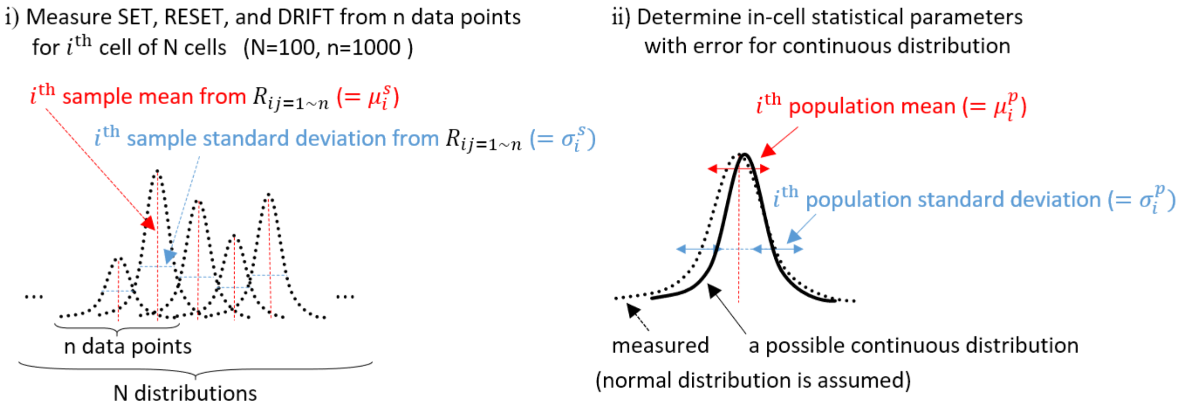

Supplementary Figure 2. The procedure i) and ii) of obtaining PCM data to build ensembles of a real PCM array. In i), $\mu_i^s = \frac{1}{n}\sum_{j=1}^{n} R_{ij}$ and $\sigma_i^s = \frac{1}{n}\sum_{j=1}^{n}\left(R_{ij} - \mu_i^s\right)^2$. In ii), $\mu_i^p = \mu_i^s + \varepsilon$, $\varepsilon = \frac{\sigma_i^p}{\sqrt{n}}$ and $\sigma_i^p = \sigma_i^s + \varepsilon$, $\varepsilon = \sqrt{\frac{2}{n-1}(\sigma_i^s)^2}$.

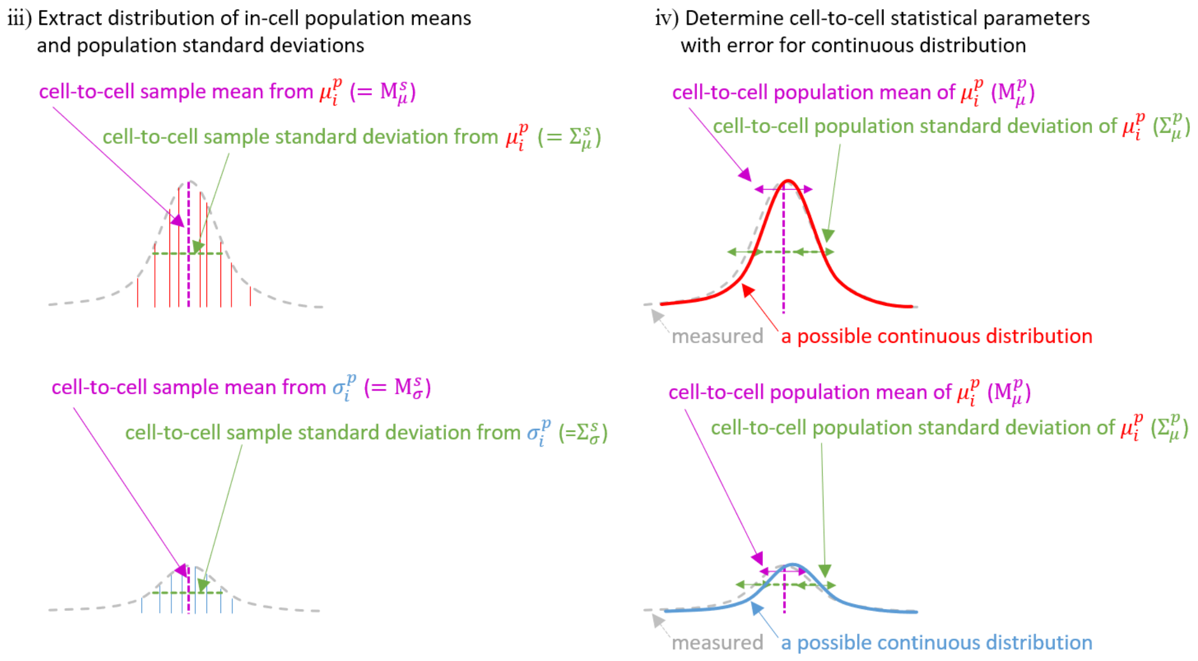

Supplementary Figure 3. The procedure iii) and iv) of obtaining PCM data to build ensembles of a real PCM array. In iii), $M_\mu^s = \frac{1}{N}\sum_{i=1}^{N}\mu_i^p$, $\Sigma_\mu^s = \frac{1}{N}\sum_{i=1}^{N}\left(\mu_i^p - M_\mu^s\right)^2$, $M_\sigma^s = \frac{1}{N}\sum_{i=1}^{N}\sigma_i^p$, and $\Sigma_\sigma^s = \frac{1}{N}\sum_{i=1}^{N}\left(\sigma_i^p - M_\sigma^s\right)^2$. In iv), $M_\mu^p = M_\mu^s + \varepsilon,\ \varepsilon = \frac{\Sigma_\mu^p}{\sqrt{N}}$ and $\Sigma_\mu^p = \Sigma_\mu^s + \varepsilon,\ \varepsilon = \sqrt{\frac{2}{N-1}(\Sigma_\mu^s)^2}$ while $M_\sigma^p = M_\sigma^s + \varepsilon,\ \varepsilon = \frac{\Sigma_\sigma^p}{\sqrt{N}}$ and $\Sigma_\sigma^p = \Sigma_\sigma^s + \varepsilon,\ \ \varepsilon = \sqrt{\frac{2}{N-1}(\Sigma_\sigma^s)^2}$

v) Sampling μ^*'s and σ^*'s

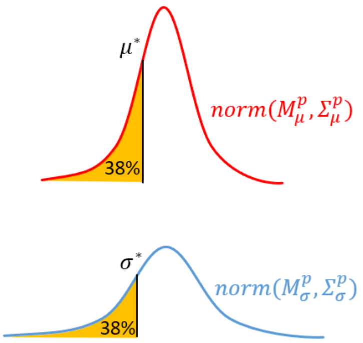

vi) μ^* and σ^* are assigned to i^{th} PCRAM for set, reset, and drift randomly.

$$PCM_i \leftarrow \left(\mu_{set}{}_i^*, \sigma_{set}{}_i^*, \mu_{reset}{}_i^*, \sigma_{reset}{}_i^*, \mu_{drift}{}_i^*, \sigma_{drift}{}_i^*\right)$$

vii) R_{set}, R_{reset}, and d_{drift} are renewed when the state is changed.

	M_μ^p	Σ_μ^p	M_σ^p	Σ_σ^p
R_{reset}	2.25×10^7	3.84×10^6	2.12×10^6	4.24×10^5
R_{set}	2.64×10^5	4.06×10^4	3.46×10^4	1.82×10^4
d_{drift}	9.29×10^{-2}	9.42×10^{-3}	4.13×10^{-4}	7.64×10^{-5}

Supplementary Figure 4. The procedure v), vi), and vii) of obtaining PCM data to build ensembles of a real PCM array. (Bottom) The table is the summary of the statistical parameters extracted from the PCM wafer. In v), $\mu^* = \mathrm{random.norm}(M_\mu^p, \Sigma_\mu^p)$ and $\sigma^* = \mathrm{norm.ppf}(\mathrm{norm.cdf}(\mu^*, M_\mu^p, \Sigma_\mu^p), M_\sigma^p, \Sigma_\sigma^p)$. In vii), $R_{set} = random.norm(\mu_{set}{}_i^*, \sigma_{set}{}_i^*)$, $R_{reset} = random.norm(\mu_{reset}{}_i^*, \sigma_{reset}{}_i^*)$, and $d_{drift} = random.norm(\mu_{drift}{}_i^*, \sigma_{drift}{}_i^*)$ for *i*-th PCM cell.

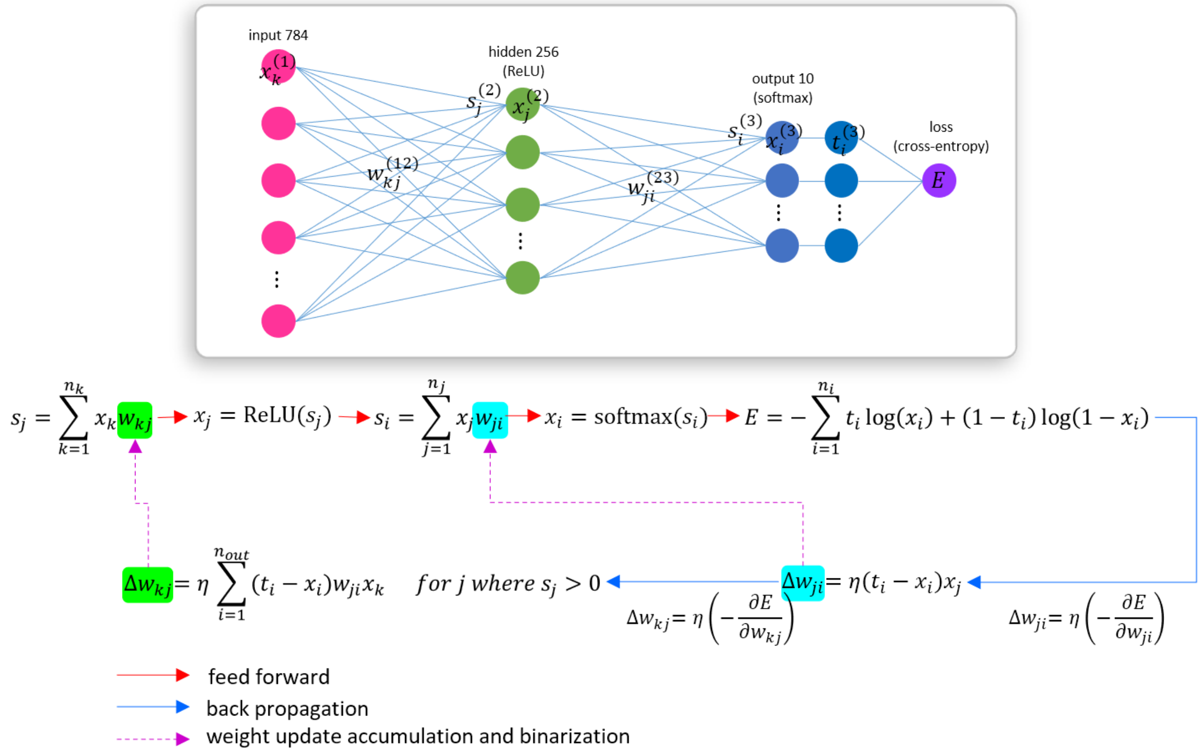

Supplementary Figure 5. The neural network model uesd in this work. s_j $(= \sum_{k=1}^{n_k} x_k w_{kj})$ is a weighted sum with conrresponding inputs (x_k) and weights (w_{kj}) in the first layer. $x_j = \mathrm{ReLU}(s_j)$ and then is used as input in the very next layer. $s_i (= \sum_{j=1}^{n_j} x_j w_{ji})$ is the weighted sum in the second layer. x_i $(= \mathrm{softmax}(s_i))$ is the final output with softmax activation. Then cross-entropy loss E $(=-\sum_{i=1}^{n_i} t_i \log(x_i) + (1 - t_i)\log(1 - x_i))=$ is obtained. $\Delta w_{ji}(=\eta\left(-\frac{\partial E}{\partial w_{ji}}\right))$ is calculated and w_{ji} is updated. Also, $\Delta w_{kj}(=\eta\left(-\frac{\partial E}{\partial w_{kj}}\right))$ is calculated and w_{kj} is updated after the binarization.

Algorithm

//Assign $\mu_{set_i^*}$, $\sigma_{set_i^*}$, $\mu_{reset_i^*}$, $\sigma_{reset_i^*}$, $\mu_{drift_i^*}$, and $\sigma_{drift_i^*}$ to all w_i^{drift}.
  for *x*= *set, reset, and drift* **do**
    for *i*=1 to *n* (number of weights) **do**
      $\mu_{x_i^*} \leftarrow random.norm\left(M_{x_\mu}^p, \Sigma_{x_\mu}^p\right)$
      $\sigma_{x_i^*} \leftarrow norm.ppf\left(norm.cdf\left(\mu^*, M_{x_\mu}^p, \Sigma_{x_\mu}^p\right), M_{x_\sigma}^p, \Sigma_{x_\sigma}^p\right)$
      $d_i \leftarrow 0$
      $t_i \leftarrow 0$
    end for
  end for
//Initialize full precision weights w_i.
  initialize(w_i)
//Implement the drift effect
  batch← 0
  loop until batch=max
    for *i*=1 to batch_size **do**
     //Binarization
      if (batch=0 and $w_i \leq 0$) or ($w_i^{old} > 0$ and $w_i \leq 0$)
        $w_i^{drift} \leftarrow random.norm(\mu_{set_i^*}, \sigma_{set_i^*})$
      else if (batch=0 and $w_i > 0$) or ($w_i^{old} \leq 0$ and $w_i > 0$)
        $w_i^{drift} \leftarrow random.norm(\mu_{reset_i^*}, \sigma_{reset_i^*})$
        $d_i \leftarrow random.norm(\mu_{drift_i^*}, \sigma_{drift_i^*})$
        $t_i \leftarrow 1 \times 10^{-6}$
      else if batch≠0 and $w_i^{old} > 0$ and $w_i > 0$
        $t_i \leftarrow t_i^{old} + 1 \times 10^{-6}$
        $w_i \leftarrow w_i^{old} + d_i \log(t_i)$
      end if
      //Inference
        ⋮
      //Error calculation
        ⋮
      //Gradient calculation
        ⋮
      //Weight update
      $w_i \leftarrow w_i^{old} + \Delta w_i$
    end for
    batch←batch+1
  end loop

Supplementary Figure 6. The pseudo algorithm to implement the consistency-induced weight increase.

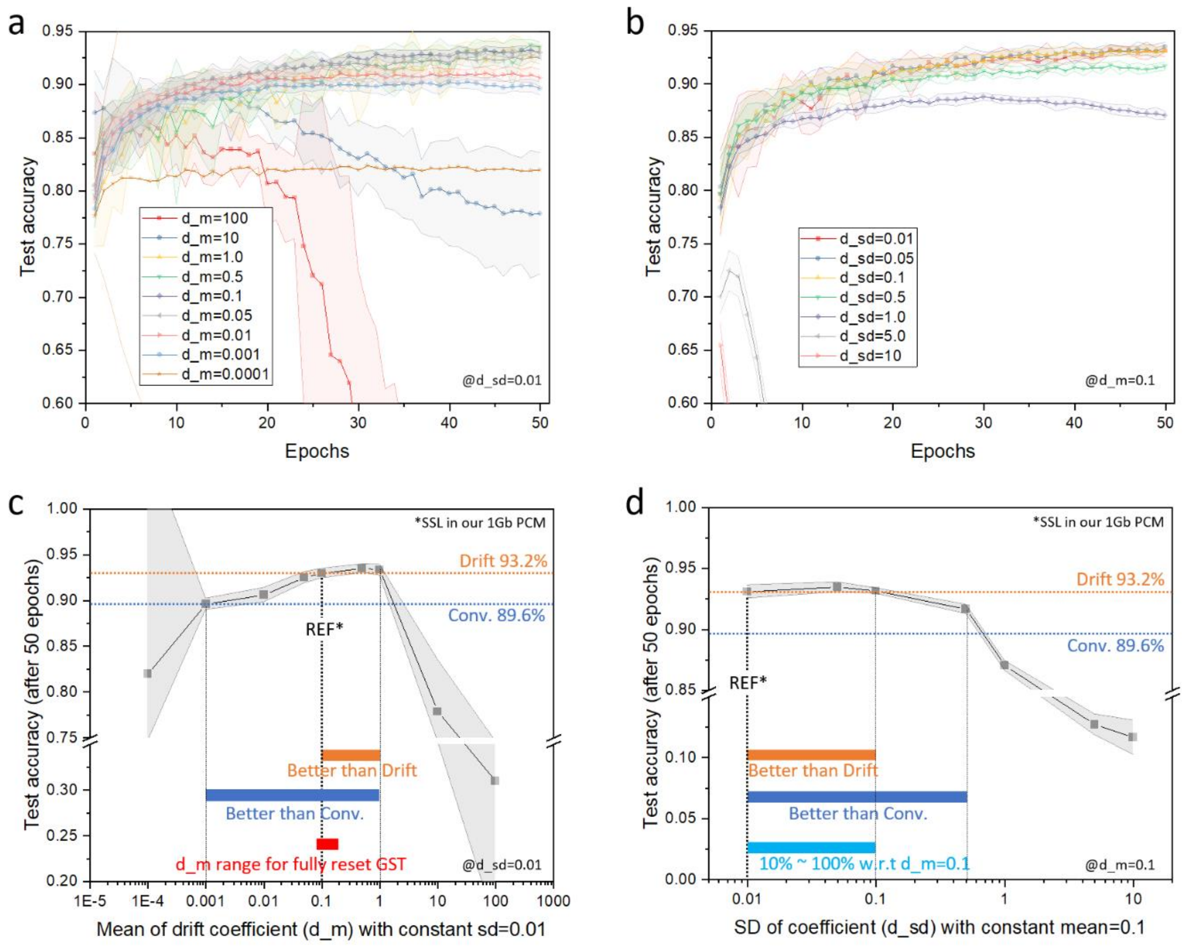

Supplementary Figure 7. The relation between the drift coefficient and the classification accuracy. The mean of drift coefficients (denoted by *d_m*) in the PCM array used is set ~0.1, and the standard deviation (denoted by *d_sd*) is ~0.01. (a) fix standard deviation (*d_sd*=0.1) and vary the mean of drift coefficients (*d_m* from 0.0001 to 100); (b) fix the mean of drift coefficients (*d_m*=0.1) and vary standard deviation (*d_sd* from 0.01 to 10), each curve represents the mean and error of the result of 10 repeats. (c) the relation between test accuracy and *d_m* after 50 epochs extracted from (a); (d) relation between the test accuracy and *d_sd* after 50 epochs extracted from (b).
